# Inflammatory Pseudotumor in the Epidural Space of Lumbosacral Spine on ^18^F-FDG PET/CT

**Published:** 2014

**Authors:** Jin-Suk Kim, Shin Young Park

**Affiliations:** 1 Department of Nuclear Medicine, Konyang University Hospital, Daejeon, South Korea; 2 Department of Pathology, Konyang University Hospital, Daejeon, South Korea

**Keywords:** Inflammatory pseudotumor, Epidural space, Lumbosacral spine, ^18^F-FDG PET/CT

## Abstract

An inflammatory pseudotumor (IPT) is a rare benign lesion, characterized by non-neoplastic proliferation of inflammatory cells and presence of intermingling collagen fibers. IPT commonly occurs in the lungs and orbita, while an intraspinal IPT is extremely rare. IPT can mimic both clinically and radiologically malignant processes, and making a definitive preoperative diagnosis is often difficult. Recently, 18-fluorine fluorodeoxyglucose (^18^F-FDG) has been reported to accumulate in IPT in the lung, spleen, liver, pancreas, colon, orbit, mediastinum, and mesentery. However, to the best of our knowledge, accumulation of ^18^F-FDG has not been reported in lumbosacral intraspinal IPT. Herein, we report a case of IPT in the epidural space of the lumbar spine, using the imaging findings of ^18^F-FDG positron emission tomography-computed tomography (PET/CT) and contrast-enhanced magnetic resonance imaging (MRI). This is the first case of IPT in the epidural space, depicted by ^18^F-FDG PET/CT, which revealed a homogeneous, intense ^18^F-FDG uptake.

## Introduction

Inflammatory pseudotumor (IPT) is a benign tumor-like lesion of unknown etiology, which has been identified in very small numbers at various locations throughout the body. It is mostly found in lungs with extrapulmonary occurrences at sites including the orbit, nasal sinuses, liver, spleen, pancreas, intestine, kidney, urinary bladder, testis, heart, and lymphatic system (1).

To date, the appearance of IPT in the spinal canal has been extremely rare. This condition has been successfully treated by surgical removal, steroid therapy, and radiation therapy to the residual mass (2). It is difficult to differentiate this pseudotumor from true neoplasm, both clinically and radiologically.

Herein, we present the first case of epidural IPT in the lumbar spine, using 18-fluorine fluorodeoxyglucose (^18^F-FDG) positron emission tomography-computed tomography (PET/CT).

## Case Report

A 43-year-old man was admitted to the hospital complaining of lower back pain with radiation to the right lower extremity. His pain had steadily worsened and he had been experiencing weakness of the right lower extremity for one and a half months; in addition, he was suffering from mild bladder dysfunction. There was no previous history of trauma or anticoagulation therapy. Hemoglobin level, white blood cell count, erythrocyte sedimentation rate, C-reactive protein level, and the results of liver function test were all normal; also, the findings on X-rays of chest and thoracolumbar spine were normal.

**Figure 1:**
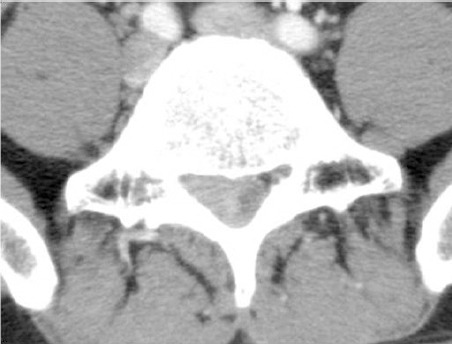
A subtly enhancing mass-like lesion in the right epidural space without bony destruction, observed on the lumbar spine CT scan

**Figure 2:**
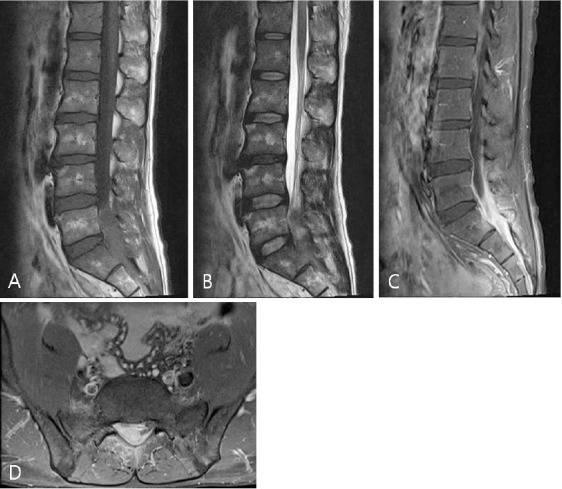
(A) MRI reveals an expansile right epidural mass from L4-5 to S2, showing homogeneous isointensity on the sagittal T1-weighted image, (B) heterogeneous iso- and hypointensity on the sagittal T2-weighted image, (C) sagittal gadolinium-enhanced T1-weighted image, and (D) axial image; a strong enhancing lesion with cord compression and abnormal signal intensity was detected in the adjacent back muscle

The lumbar spine CT scan showed a homo-geneous mass-like lesion with enhancement in the right epidural space (Figure 1). Magnetic resonance imaging (MRI) demonstrated an epidural mass from L4-5 to S2, compressing the thecal sac, and extending to the right epidural space along the nerve root. It involved homogeneous isointensity on the T1-weighted image, heterogeneous iso- and hypointensity on the T2-weighted image, and strong enhancement (Figure 2). MRI also showed abnormal signal intensity in the adjacent back muscle.

**Figure 3:**
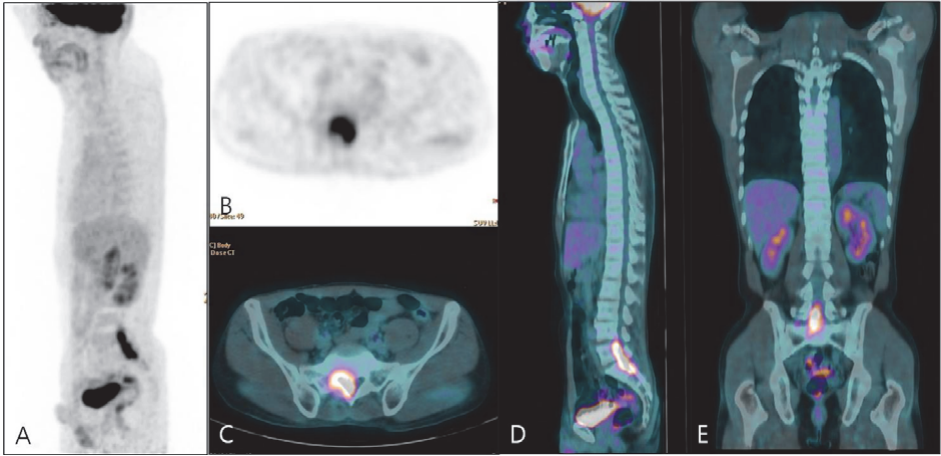
^18^F-FDG sagittal maximum intensity projection (MIP, A), axial PET (B), axial PET/CT (C), sagittal PET/CT (D), andcoronal PET/CT (E) images demonstrated intense FDG uptake (SUV_max_ 6.7), corresponding to the right spinal canal of the lumbosacral spine; there was no abnormal FDG uptake in other regions

There was no evidence of adjacent bone destruction or bony sclerosis on CT or MRI images. The differential diagnoses included spinal epidural malignancies, such as spinal lymphoma, metastatic tumor, or epidural abscess. The patient underwent ^18^F-FDG PET/CT(Gemini TF, Philips Healthcare, OH, USA) for the detection of primary cancer and metastatic disease. The imaging showed intense uptake of FDG with maximal standardized uptake value (SUV_max_) of 6.7 in the right epidural space (Figure 3). There was no abnormal FDG uptake in other organs except the spinal canal. This finding suggested that a primary tumor rather than metastasis originated in the spinal canal.

**Figure 4:**
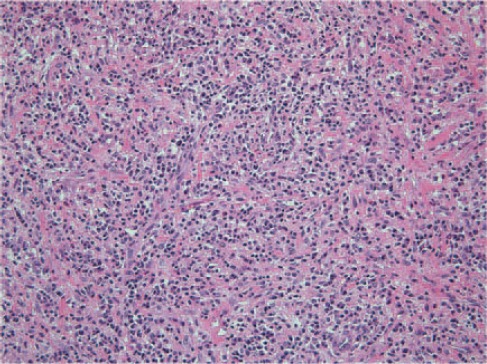
Microscopically, the epidural mass showed extensive infiltration of lymphocytes, neutrophils, and fibrosis (H&E, ×200)

We performed an L5-S1 hemilaminectomy and microscopic subtotal resection of the mass, which was located in the epidural space inside the ligamentum flavum. The mass was highly hypervascular and firmly attached to the dura, the ligamentum flavum, and the bone. Histologically, the tumor consisted of infiltrated inflammatory cells including lymphoplasma cells, neutrophils, and eosinophils in a background of stroma, composed of myofibroblasts and collagen bundles (Figure 4).

Immunohistochemical staining showed < 2 IgG4-positive plasma cells per high power field, and the tumor was diagnosed as an inflammatory pseudotumor. After surgery, the patient started steroid therapy and his pain and neurologic symptoms steadily improved.

## Discussion

IPT, which is synonymous with inflammatory myofibroblastic tumors, is a rare benign tumor with unknown pathogenesis. Clinical features and imaging findings of IPT are similar to those of malignant tumors. This tumor consists of a background proliferation of spindle-shaped mesenchymal cells, associated with a variable infiltration of inflammatory cells.

IPT most commonly involves the lungs and orbita, but it has been reported to occur in nearly every site in the body (3). Although the central nervous system is the rarest site affected by IPT, more than 100 sporadic cases have been reported in the literature (4, 5). Intraspinal IPTs are extremely rare. Except for the present case, we only found seven previous reports of epidural IPT in the spine, and all of them were located in the cervical or thoracic spine (1, 6-10) (Table 1). Furthermore, these cases were not described using the ^18^F-FDG PET/CT findings.

**Table 1:** Previous reports of cases with epidural inflammatory pseudotumors in the spine

Source	Age (y)/sex	Location	Co-morbidity
**Roberts et al., 1997 (1)**	58/F	T9-T11	Hypertension
**Gilliard et al., 2000 (6)**	45/M	C3-T2	Multifocal fibrosclerosis
**Roberts et al., 2001 (7)**	39/F	T5-T6	None
**Seol et al., 2005 (8)**	44/M	T1-T7	Not reported
**Sailler et al., 2006 (9)**	78/M	C6-T3	Giant cell arteritis
	73/F	T5-T7	Giant cell arteritis
**Kato et al., 2012 (10)**	63/M	T5-T6	Polymyalgia rheumatica
**The present case**	43/M	L4-S2	None

The current case is the first report of an epidural IPT in the lumbosacral spine, which showed a high uptake of FDG, as detected by ^18^F-FDG PET/CT. The clinical and imaging characteristics of IPT are non-specific, since the lesion has a wide range of clinical and imaging presentations. Hence, the diagnosis of IPT can be made only after other specific disorders are ruled out. Commonly, the differential diagnoses considered in spinal IPT cases include spinal lymphoma, metastatic tumor, and multiple myeloma (8).

Radiologically, IPT appears as a solid intraspinal tumor. On MRI images, IPT usually has a low signal intensity on both T1- and T2-weighted images, which may reflect the fibrotic nature of these lesions. Contrast-enhanced MRI may show a homogeneous or heterogeneous lesion and delayed imaging often shows increasing enhancement due to the presence of fibrosis (11).

To our knowledge, there have been no reports on intraspinal IPT with ^18^F-FDG PET/CT. However, as in our case, marked increase in FDG uptake has been already reported as a feature of IPT (12-16). It is well established that FDG uptake is not specific to malignant neoplasms, and it may be observed in a variety of tissues with increased glucose consumption.

The mechanism of high FDG uptake in IPT may be related to inflammatory cells in the pseudotumor (16). As neutrophils were found in the tumor based on the histopathological findings in this case, their presence may contribute to the mechanism of FDG uptake in IPT.

It is impossible to distinguish malignant neoplasms from inflammatory pseudotumors based on imaging findings of MRI and ^18^F-FDG PET/CT; therefore, without a biopsy, making a differential diagnoses is very difficult. However, ^18^F-FDG and ^18^F-FDG PET/CT have been reported to be useful in evaluating the therapeutic effects of steroid or radiation therapies on IPT in cases with incomplete surgical resection (17, 18).

Surgical excision is usually mandatory in IPT, compressing the spinal cord, due to the emergent need to relieve the mass effect; it is generally curative when total excision is performed. Radiotherapy, systemic steroid, or immunosuppressive drugs are also administered for IPT patients, which may lead to a decrease in the mass volume (6, 19, 20).
